# Development and characterization of an intra-articular fracture mediated model of post-traumatic osteoarthritis

**DOI:** 10.1186/s40634-023-00625-9

**Published:** 2023-07-04

**Authors:** Michael S. Valerio, William A. Pace, Connor P. Dolan, Jorge B. Edwards, Naveena B. Janakiram, Benjamin K. Potter, Christopher L. Dearth, Stephen M. Goldman

**Affiliations:** 1 Research and Surveillance Division DoD-VA Extremity Trauma and Amputation Center of Excellence, Bethesda, USA; 2 Department of Surgery, Uniformed Services University of the Health Sciences-Walter Reed National Military Medical Center, Bethesda, USA; 3 Department of Biomedical Engineering, Vanderbilt University, Nashville, USA; 4 Department of Veterinary Physiology & Pharmacology, Texas A&M University, College Station, TX, USA; 5 The Henry M. Jackson Foundation for the Advancement of Military Medicine, Bethesda, USA; 6 Translational Research Program, Division of Cancer Treatment and Diagnosis (DCTD), National Cancer Institute (NCI), Rockville, MD USA; 7 Department of Orthopedic Surgery, Walter Reed National Military Medical Center, Bethesda, USA

**Keywords:** Animal Models, Musculoskeletal System, Trauma, Comminuted Fractures, Military Personnel, Regenerative Medicine

## Abstract

**Purpose:**

This study aimed to develop and characterize a closed intra-articular fracture (IAF) mediated post-traumatic osteoarthritis (PTOA) model in rats to serve as a testbed for putative disease modifying interventions.

**Methods:**

Male rats were subject to a 0 Joule (J), 1 J, 3 J, or 5 J blunt-force impact to the lateral aspect of the knee and allowed to heal for 14 and 56 days. Micro-CT was performed at time of injury and at the specified endpoints to assess bone morphometry and bone mineral density measurements. Cytokines and osteochondral degradation markers were assayed from serum and synovial fluid via immunoassays. Histopathological analyses were performed on decalcified tissues and assessed for evidence of osteochondral degradation.

**Results:**

High-energy (5 J) blunt impacts consistently induced IAF to the proximal tibia, distal femur, or both while lower energy (1 J and 3 J) impacts did not. CCL2 was found to be elevated in the synovial fluid of rats with IAF at both 14- and 56-days post-injury while COMP and NTX-1 were upregulated chronically relative to sham controls. Histological analysis showed increased immune cell infiltration, increased osteoclasts and osteochondral degradation with IAF relative to sham.

**Conclusion:**

Based on results from the current study, our data indicates that a 5 J blunt-forced impact adequately and consistently induces hallmark osteoarthritic changes to the articular surface and subchondral bone at 56 days after IAF. Marked development of PTOA pathobiology suggest this model will provide a robust testbed for screening putative disease modifying interventions that might be translated to the clinic for militarily relevant, high-energy joint injuries.

**Supplementary Information:**

The online version contains supplementary material available at 10.1186/s40634-023-00625-9.

## Introduction

Musculoskeletal injuries to the extremities account for more than half of all combat wounds sustained by US Service members (SM) in the most recent military conflicts [1]. Besides conflict induced injuries, SM are also exposed to increased incidence of activity or training-based injuries (e.g. parachuting out of planes, drops from heights, etc.). In the event that such injuries affect a synovial joint, damage to the articular surface often occurs either through direct insult or indirectly as a result of subsequent incongruities and altered loading [2, 3]. This damage may take the form of fissures, flaps, and tears, which are permanent—due to the limited endogenous regenerative capacity of articular cartilage. Injuries that penetrate to the subchondral bone (e.g. intra-articular fractures, IAF) often result in a robust inflammatory response that is associated with fibrocartilage deposition within the osteochondral defects [4, 5]. These sites subsequently result in a high reoccurrence of injury and progressive degeneration through a process termed post-traumatic osteoarthritis (PTOA). PTOA is a debilitating condition that accounts for approximately 12% of the $100 billion annual socioeconomic burdens of the more broadly defined osteoarthritis (OA) and is known to be associated with chronic pain, reduced mobility, and other deleterious impacts on quality of life [6]. Unfortunately, PTOA is such a pervasive component of the longer-term sequelae of combat-related injuries that it has been identified as the most prominent deleterious secondary health condition which results in the medical discharge of active duty SMs [1]. While older patients with idiopathic OA can often be treated with joint replacement or joint fusion, these approaches are suboptimal for the younger and physically active populations such as combat-wounded SMs. Younger populations that experience IAF face the prospect of dealing with life altering symptoms for multiple decades and would benefit from interventions that might prevent the onset and/or mitigate the progression of PTOA. Unfortunately, such disease modifying interventions are not readily available and will require further investigation and evaluation to develop.

Animal models of PTOA are frequently used in the preclinical development of putative disease modifying interventions due to their ability to quickly and reliably induce osteoarthritic pathobiology in the joint. Many of the standard surgically induced PTOA models (e.g., ACL transection [7], destabilization of the medial meniscus [8] or medial meniscus transection [9]), to name a few, however induce more mild lesions that might not fully recapitulate the pathobiology of more severe high-energy injuries that penetrate the subchondral bone. Moreover, the currently available non-invasive IAF models (e.g. fracture indenter model [10, 11]) are based on highly localized, displacement-controlled insults which may not disturb the surrounding soft tissue nor recapitulate the high-energy nature of combat-related trauma.

One approach to better recapitulate the effects of high-energy trauma is through blunt impact loading as is represented by a small number of reports in the literature. Specifically, Seol et al. [12] utilized a drop tower in mice finding that impacts on the order of 100 mJ induced subtle osteoarthritic changes characterized by surface and mild proteoglycan depletion, while Swart et al. [5] used a similar approach in rats, but focused only on acute outcomes (≤ 72 h) associated with chondrocyte death and did not fully investigate the progression of injury to assess pathological changes; neither of these models reproduced the high-energy lower extremity trauma (HELET) associated with severe combat-related (or civilian) injuries. Therefore, an opportunity exists to build upon the prior investigations and develop a non-invasive rat model of IAF and characterize the degenerative changes associated with PTOA at later time points. As such, the objective of the current study was to develop a militarily relevant model of IAF-induced PTOA that can be used as a robust testbed for the evaluation and further translation of putative disease modifying interventions for PTOA. Our hypothesis is that inducing high energy impacts, like those incurred in traumatic clinical scenarios, will results in IAF that will develop chronic osteoarthritic pathology, and that these injuries would further result in detectable and, potentially, enduring changes in synovial and systemic biomarkers.

## Methods

### Animals

This study was conducted in compliance with the Animal Welfare Act and in accordance with both the Animal Research: Reporting of In Vivo Experiments (ARRIVE) guidelines and the principles of the Guide for the Care and Use of Laboratory Animals. All animal procedures were approved by the Institutional Animal Care and Use Committee (Protocol: SUR-19–966) and conducted in the AAALAC accredited facilities. The corresponding ARRIVE checklist is included in the supplementary material. Skeletally mature male Lewis rats (> 10 Weeks, 337.3 g ± 31.9 g, *N* = 36) were maintained on a 12-h light–dark schedule in standard large rodent cages with ad libitum access to food and water. Laboratory animals were euthanized at predefined endpoints of 14 and 56 days via intra-cardiac delivery of a lethal dose of sodium pentobarbital while under isoflurane anesthesia.

### Induction of intra-articular fracture

**Fig. 1:**
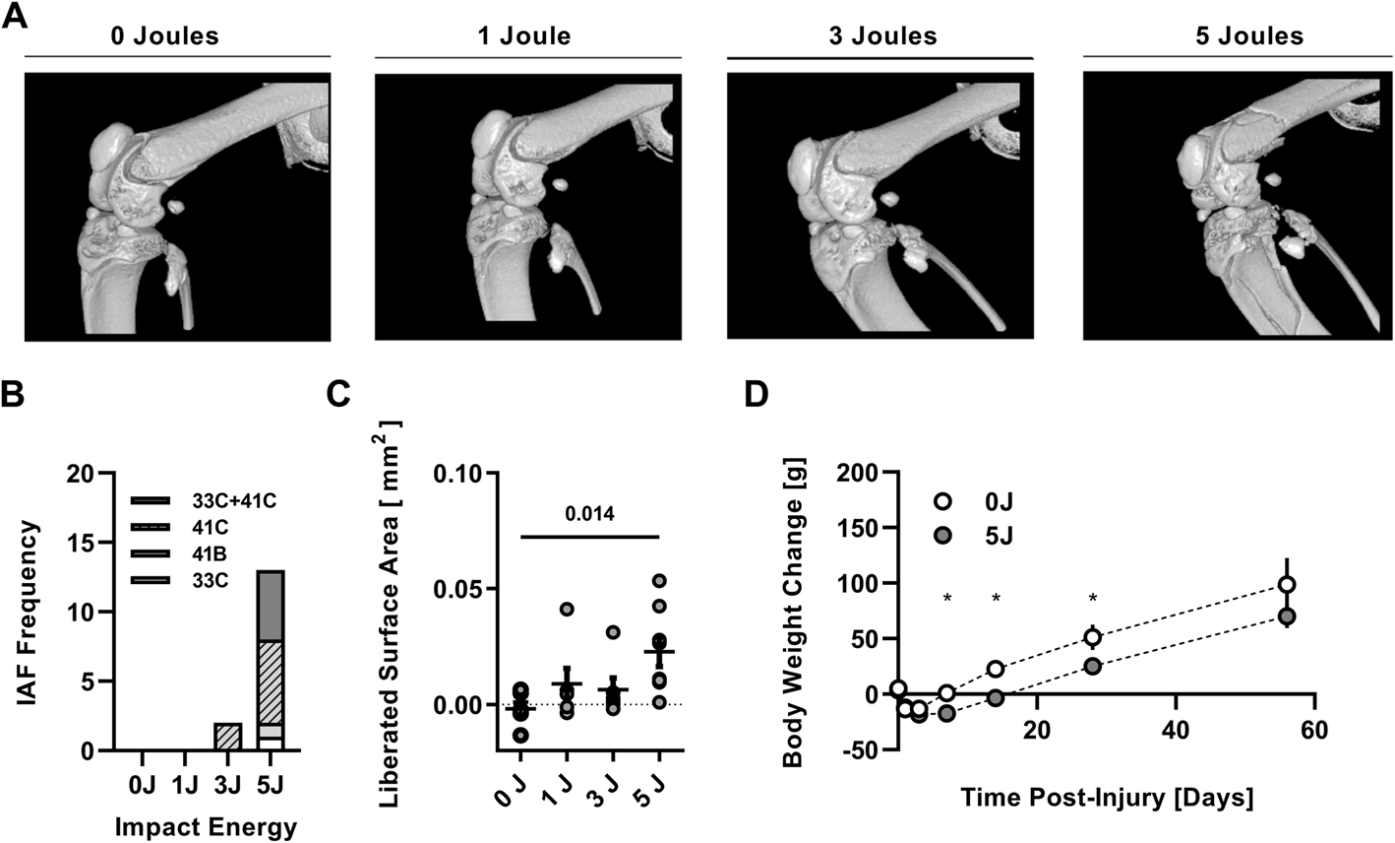
Validation of IAF-induction. **A** Representative 3D rendered images of micro-CT scans immediately following injury show multifactorial fractures extending into the intraarticular surface. **B** Fracture frequency based on 2018 OTA fracture classification compendium. **C** Liberated surface area of tibia/fibula (top) or femur (bottom) in response to sham (0 J), 1 J, 3 J or 5 J impact energies. **D** Body weight change over time, normalized to pre-injury weight (grams) Values are representative images or mean ± SD

Rats (*n* = 6/group) were subjected to general anesthesia (2–4% inhaled isoflurane in oxygen) and were given a one-time injection of buprenorphine slow-release (SR) at a concentration of 1 mg/kg body weight. Animals were then randomly allocated to 4 experimental groups differentiated based on the amount of impact energy to be delivered to the knee: 0 J (Sham), 1 J, 3 J, or 5 J. Impacts were subsequently delivered to the lateral aspect of the left knee using a drop tower loaded with a 2 kg slug and depicted in Supplemental. Fig. 1. Once a repeatable impact energy was identified, two loading groups were excluded from further analysis and longer term experiments were initiated. Due to the nature of the animal model, investigators were unblinded at the time of IAF induction, but were otherwise blinded to study group at all subsequent data collections.

### Sample collection

Whole blood was collected from the tail artery prior to and immediately following knee impacts. Subsequent blood draws were also performed at 1, 3, 7, 14, and 56-days post-injury. After collection, blood was allowed to clot and serum was isolated by centrifugation at 1000RPM for 10 min at room temperature. At the terminal endpoints, knee joint synovial fluid (SF) was collected by accessing by transecting the patellar tendon distally and cutting a small window into the synovium. Whatman paper was placed into the cavity to absorb the synovial fluid until saturated. All samples were snap frozen in liquid nitrogen and stored at -80 °C for subsequent analysis. Rat hindlimbs were subsequently disarticulated at the hip and fixed in 10% neutral buffered formalin.

### Micro-computed tomography

Pre- and post-injury micro-computed tomography (μ-CT) was performed to assess fracture severity and assessments were taken at 14 and 56 days to assess healing (SkyScan 1278, Bruker Corporation). Animals were placed under isoflurane anesthesia and lower limbs were aligned for imaging using a custom frame. Scans were conducted at a 50-micron voxel size using a 1 mm aluminum filter, with a voltage of 65 kV, current of 770uA, and 56 ms exposure with 360° of rotation in 0.3^°^ increments. Each scan lasted approximately 10 min and yielded 400 milligreyes (mGy) of radiation. Three dimensional images were rendered from the reconstructed scans and grey values were thresholded for visualization and quantitative assessment. Regions of interest comprised of the epiphyseal and metaphyseal spaces were contoured for bone morphometry (BMM) and bone mineral density (BMD) measurements. For isolation of the femoral and tibial epiphyses, transaxial sections were used to determine landmarks and marked as slice 1, and moving proximally 30-slices (150 µm) or from the proximal tibia and 10 slices (50 µm) distally, were used to isolate the epiphyseal sections.

### Fracture assessment

3D reconstructions derived from micro-CT scans were used to score fractures based on the 2018 Fracture Classification and Dislocation Compendium produced by the AO Foundation and Orthopaedic Trauma Association [13]. Five images representing a 360-degree view of the injured and contralateral limbs were generated. A single score was generated based on the majority decision of a group of seven independent raters who were blinded to identifying information about the samples. The frequency of fractures classes was noted for both the femur and tibia from each sample and tallied. Additionally, quantitative analysis of bone surface area was performed on the two-dimensional slices of micro-CT scans at the pre-injury and immediate post-injury time points to determine the bone surface area liberated by the impact [14].

### Synovial fluid protein extraction

Frozen SF samples collected with Whatman paper were obtained from storage and were reconstituted in 300ul of cell lysis buffer (Catalog # EPX-99999–000, ThermoFisher) overnight at 4 °C with gentle agitation. 100 µl of the extracted sample was then removed and placed into a collection tube for each appropriate sample. The paper and fluid were then transferred to tissue homogenization tubes (Catalog # 116,910,050-CF, MP Biosciences) and pulverized using a tissue homogenizer at the highest setting for 20 s. The remaining Whatman paper was removed via a filtration step and samples were aliquoted for downstream immunoassays.

### Protein analysis

Serum samples and SF extracts were analyzed using a cytokine and chemokine multiplex immunoassay according to the manufacturer’s protocol and recommendations (Catalog # EPX220-30,122–901, ThermoFisher). Briefly, serum and SF samples were thawed on ice. Protein-specific microbeads were distributed evenly between all wells. Assay loading buffer was added to each well, followed by standards and samples to appropriate wells for 90 min, followed by wash steps, secondary antibody capture for 60 min, more washes, and fluorescence tagging. Multiplex plates were read on a BioRad200 Multiplex Plate Reader using the Bio-Rad software according to the manufacturer’s recommendations. Raw data was extracted via software based on the input of assay standards and dilution factors to calculate sample values. Samples found to be below the detectable limit were assigned a value of one-half of the lower limit of detection of the assay. Results are only presented for analytes that exhibited measurable levels of the target in > 80% of the samples tested.

In addition to multiplex assays, SF samples were obtained to measure urea as a biochemical marker to normalize protein data. Additionally, serum from Day 56 endpoints was used to measure markers of bone and cartilage breakdown. Rat-specific sandwich ELISA kits for, Urea (Catalog # MBS2600001, MyBioSource) COMP (Catalog # MBS2512987, MyBioSource) and NTX1 (Catalog # MBS4501525, MyBioSource) were run according to manufacturer’s specifications. Absorbance values were obtained via a BioTek plate reader at 450 nm and values were calculated relative to linear standards.

### Histological and immunohistochemical analysis

Whole hindlimbs were resected from each animal and placed in 10% neutral buffered formalin for fixation. Hindlimbs were subsequently rinsed in saline and decalcified with Cal-EX™ solution (Catalog # CS510-1D, ThermoFisher) for 14 days. Decalcified samples were embedded in paraffin blocks for fixation and sectioning. Coronal sections were cut to a thickness of five microns and stained with hematoxylin and eosin (H&E) for histologic analysis. To evaluate synovitis, H&E sections were used to evaluate vascularity (0–2), Detritus (0–2) and Fibrosis (0–3) as previously described [15]. In addition to H&E, sections were stained for presence of osteoclasts (OC) at Day 14 and Day 56 post-injury using the OC surface marker DC-STAMP. Briefly, decalcified and rehydrated samples were blocked with 5% normal goat serum (NGS) for 1 h then incubated with DC-STAMP (Catalog # ab238151, Abcam) diluted 1:50 with NGS. Following incubation, slides were incubated in goat-anti-rabbit-HRP secondary (Catalog # ab214880, Abcam) and then developed with DAB substrate (Catalog # SK-4105, Vector labs).

### Statistical analysis

Study outcomes were compared by analysis of variance (ANOVA) with Holm-Šídák post-hoc tests and are reported as the mean ± SD with a significance level set at α = 0.05 (Graphpad Prism 9.4). Samples sizes were determined by a power analysis to detect an effect size of 0.7 using a Chi-squared test of IAF frequency with 4 groups, power of 0.80, and alpha of 0.05. Knowing experimental groups would be downselected to one repeatable impact energy for subsequent analyses, we verified this sample size would be sufficient for detecting a difference in biochemical and imaging-based study outcomes (effect size 0.6, α = 0.05, β = 0.80) using a power analysis designed for a fixed effects ANOVA (GPower 3.1.9.2) with 4 groups and 1 degree of freedom.

## Results

### Intra-articular fracture outcomes of impact loading

Immediate post-injury, micro-CT imaging revealed fracture outcomes varied qualitatively with increased severity with higher impact energy (Fig. 1A). Scoring of micro-CT renderings showed the 5 J impact group was characterized by a high frequency of type C (fragmentary and comminuted) fractures to both femurs and tibias (Fig. 1B) while no major fractures were produced in the 1 J cohort and an intra-articular fracture of the femur or tibia was produced in only 2 of 6 rodents in the 3 J cohort. Quantitative analyses concur with the qualitative scoring outcomes as the liberated epiphyseal bone surface area was increased in only the 5 J impact group relative to sham (0 J) controls (Fig. 1C). Based on these outcomes, the 1 J and 3 J group were discontinued from further analysis as IAF induction in this group was not reliable.

### Gross physiological response to injury

Body weights were found to vary slightly between experimental groups as a function of time since injury (Interaction: Group x Time, η^2^ = 0.04, *P* < 0.05) (Fig. 1D). Specifically, the weights of the animals from the high energy group (5 J) were the same as the sham controls for 7 days post-injury, but then gained less weight for the remainder of the experimental time course.

### Soluble factor analysis

**Fig. 2:**
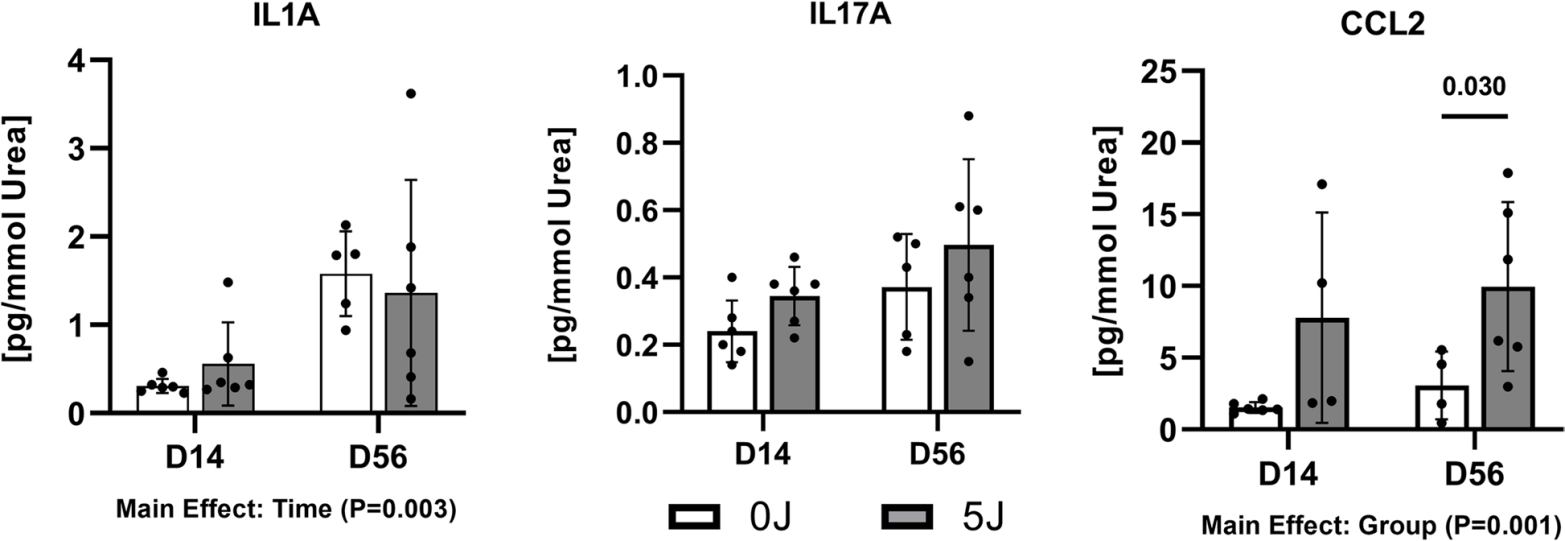
Soluble Factor Analysis from Synovial Fluid Extracts. Pro- and anti-inflammatory cytokines at Day 14 and Day 56. All samples are individually normalized to urea content. Values are represented as individual data points and mean ± SD

Serum levels of a myriad of cytokines and chemokines varied over the experimental time course and between groups (Fig. 2). Of particular interest, IL17A (Main Effect, *P* < 0.001) and CXCL1 (Main Effect, *P* < 0.001) expression was reduced in the 5 J group relative to the 0 J group throughout the experimental timecourse. Other factors including CCL2, CCL3, CCL11, CXCL2 (MIP-2α), and CCL7 exhibited disparate expression between groups as a function of time since injury (Interaction, *P* < 0.05). At one day post-injury, CCL2 (*P* < 0.001) and CCL7 (*P* < 0.001) were both elevated in the 5 J group relative to the 0 J controls. Subsequently, by 7 days post-injury CXCL2 was found to be more highly expressed in the 5 J group (*P* = 0.048) and remained elevated relative to the 0 J controls at 14 (*P* = 0.005) and 56 (*P* = 0.028) days post-injury. CCL2 (*P* < 0.001) and CCL11 (*p* = 0.044) were found to be decreased at 56 days post-injury in the 5 J group relative to 0 J.

**Fig. 3:**
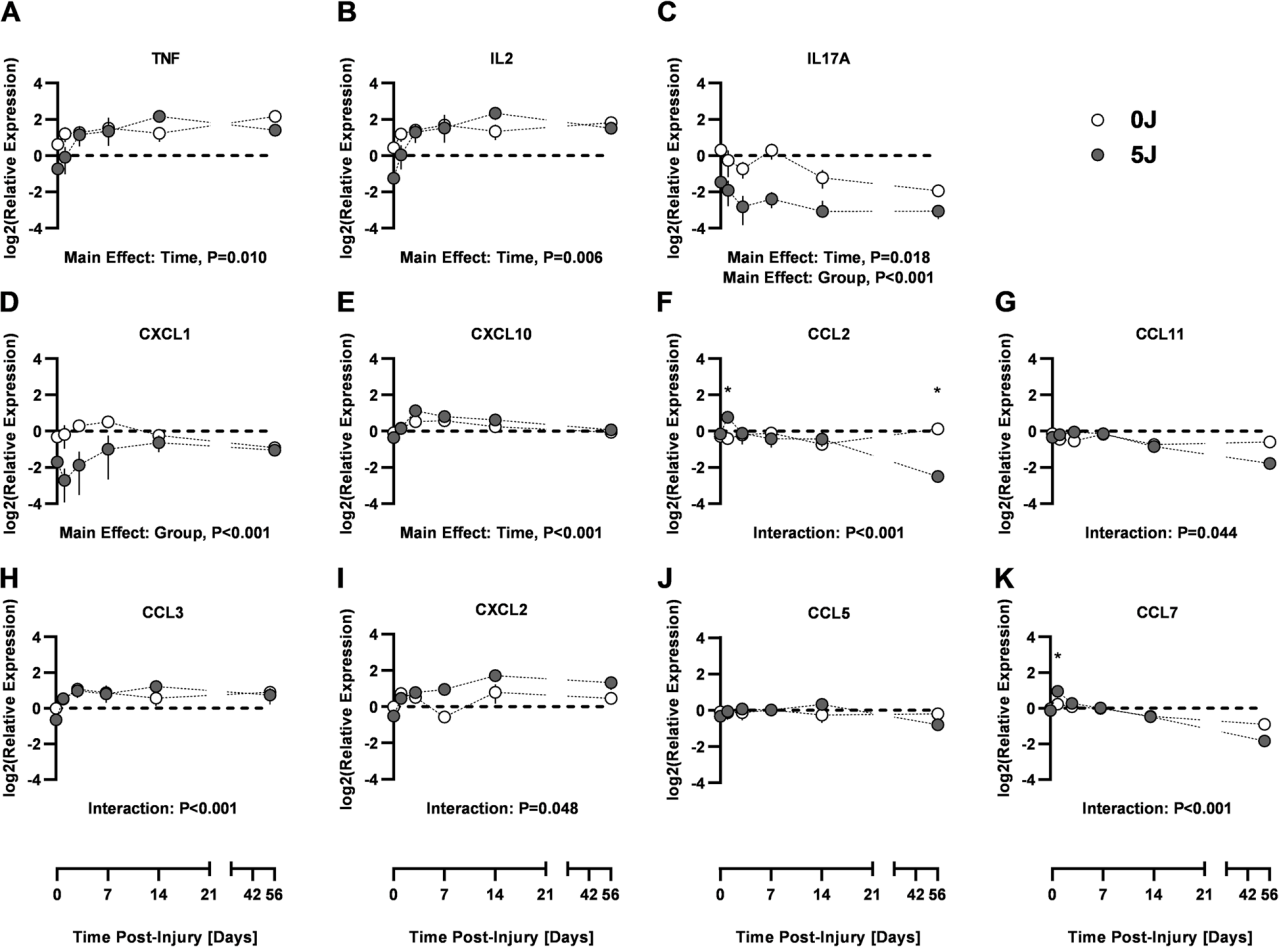
Soluble Factor Analysis from Serum. Pro- and anti-inflammatory cytokines levels are presented at Days 0, 1, 3, 7, 14, and Day 56 post-injury as the base 2 logarithm of expression relative to the pre-injury baseline. Values are mean ± SD. Dashed line and shading represent the mean ± SD of the pre-injury baseline. Main Effects are listed according to significant changes per group, time specific changes are marked where * (*P* < 0.05)

The observed systemic cytokine and chemokine response was coupled with local alterations in soluble factor profiles within the SF of the affected joints (Fig. 3). CCL2 expression, in particular, was increased in the SF between the experimental loading conditions across study endpoints relative to the sham control group (Main Effect, *P* = 0.001). IL1A exhibited increased expression as a function of time in both groups, but no difference between groups was observed. All other cytokines assayed were either not differentially expressed at either time point or expressed at a level that was below the limit of detection.

**Fig. 4:**
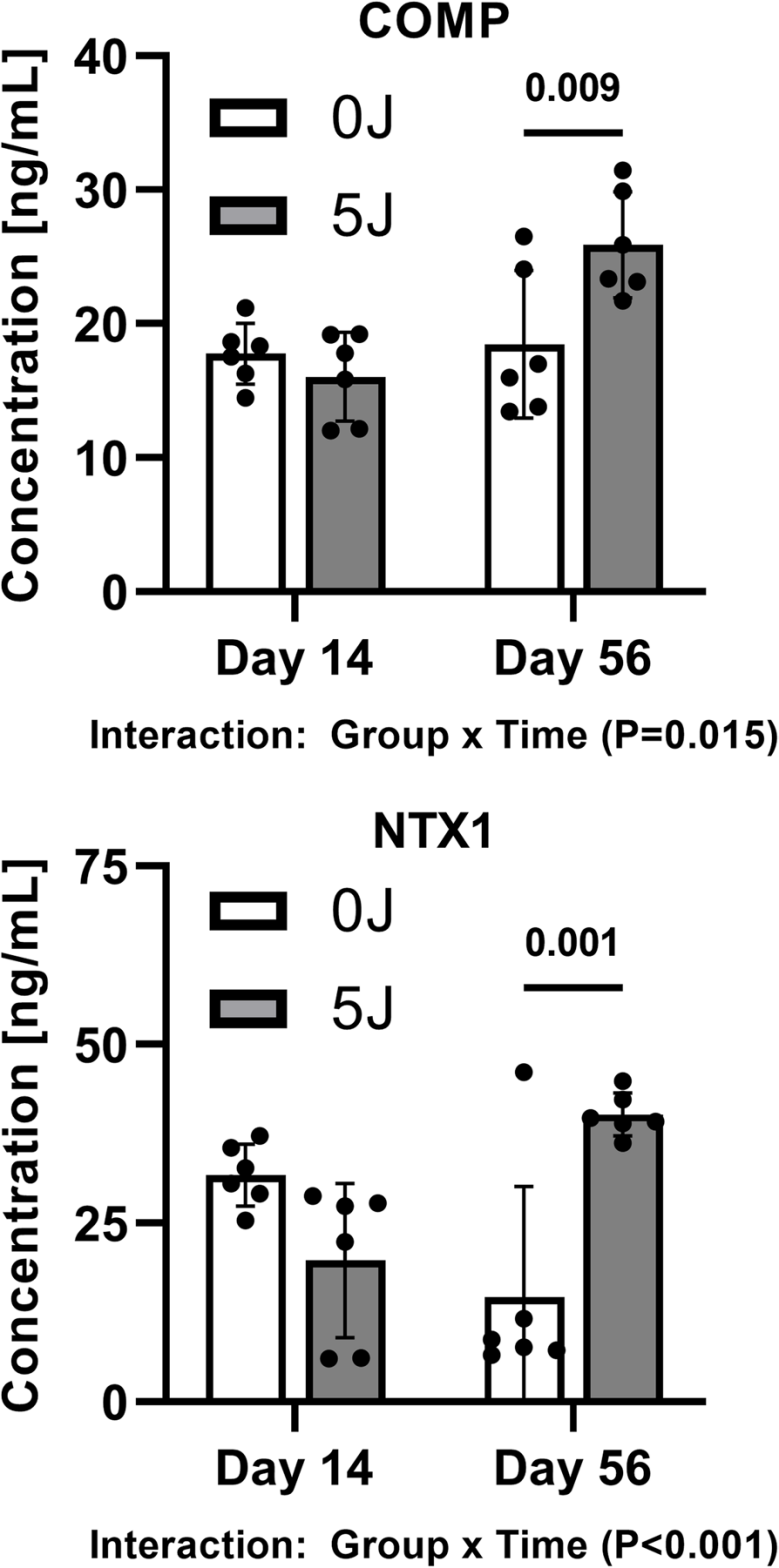
Osteochondral Degradation Marker Analysis. COMP and NTX1 expression from serum collected from sham (0 J) and 5 J injury groups at Day 14 and Day 56 post-injury. Values are represented as individual data points and mean ± SD

In addition to measuring systemic and local inflammatory markers, COMP and NTX1 were assessed as systemic markers of cartilage and bone turnover, respectively. Neither marker (Fig. 4) was not found to vary between groups at 14 days post-injury, but both COMP (*P* = 0.009) and NTX-1 (*P* = 0.001) were found to be disparately elevated in the 5 J group relative to the 0 J controls.

### Micro-computed tomography analysis

**Fig. 5:**
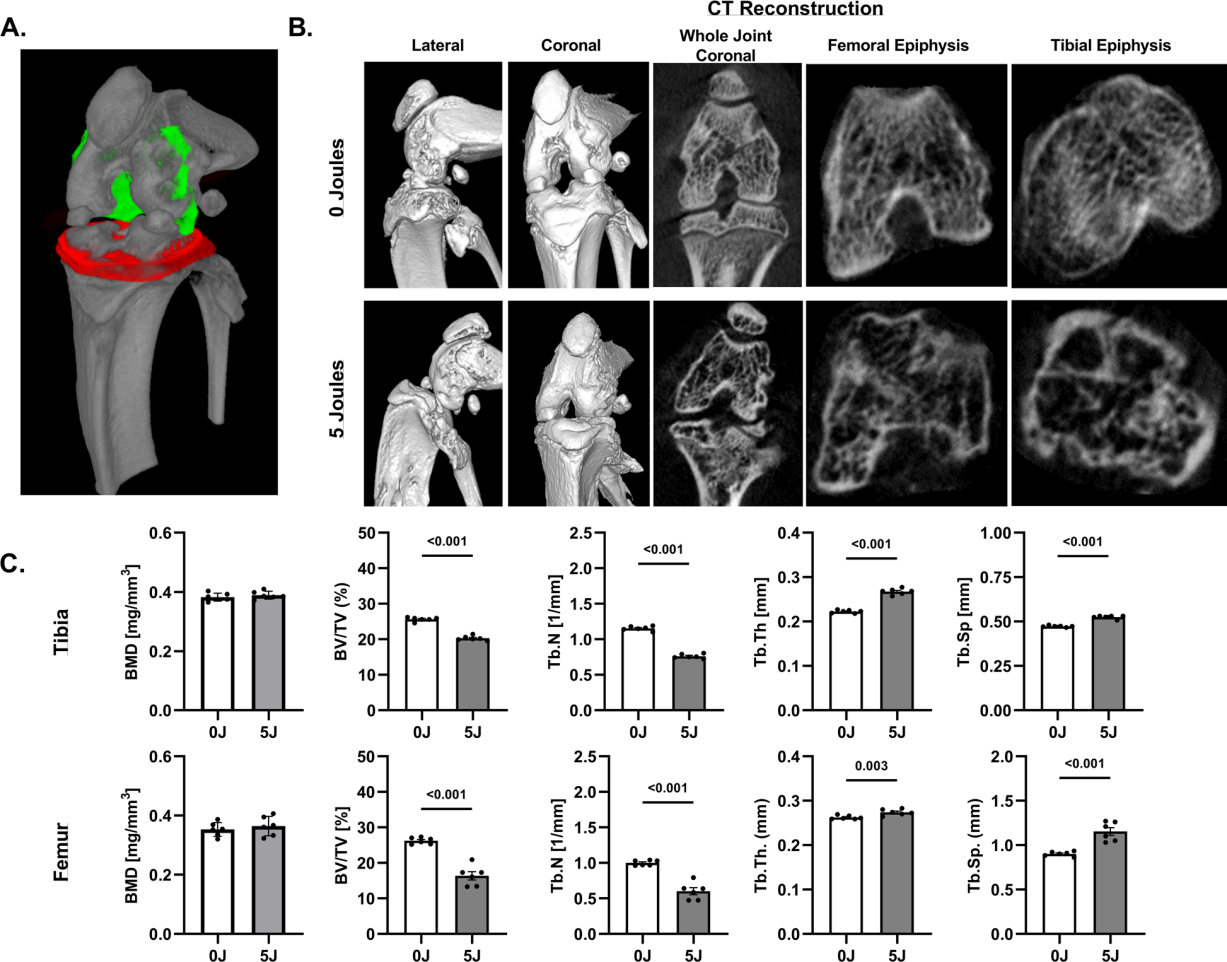
Quantitative Micro-CT Assessment of IAF Healing. **A** Representative micro-CT reconstructions and transaxial slices through the femoral and tibial epiphyses of sham (0 J) and 5 J impact groups 56 days post-injury. **B** Bone mineral density and morphometry measurements for the tibial and femoral epiphyseal regions exhibit chronic (Day 56) changes in the arrangement of the subchondral bone architecture. Values are represented as individual data points and mean ± SD

Reconstructed micro-CT scans and whole, coronal and transaxial sections through the tibial and femoral epiphysis reveal fracture healing differences owing to IAF (Fig. 5A). These data were used to measure BMD and BMM at the terminal point of healing following PTOA-induced injury. While no differences in BMD were observed, there were stark differences in BMM of the tibial plateau (epiphysis) between the experimental groups (Fig. 5B). Specifically, bone volume (BV/TV) and trabecular number (Tb.N) were reduced in the 5 J group compared to 0 J (*P* < 0.001), while trabecular thickness (Tb.Th) and trabecular spacing (Tb.Sp) were increased (*P* < 0.01) in both the tibial and femoral epiphysis. Reconstructed and coronal CT images (Supplemental Fig. 2) compiled from Day 0, 14 and 56 post-injury for 0 J and 5 J groups showed a marked increase in osteophytes as well as bone and joint degradation over time, thus supporting the BMM results.

### Histological and immunohistochemical analysis

**Fig. 6:**
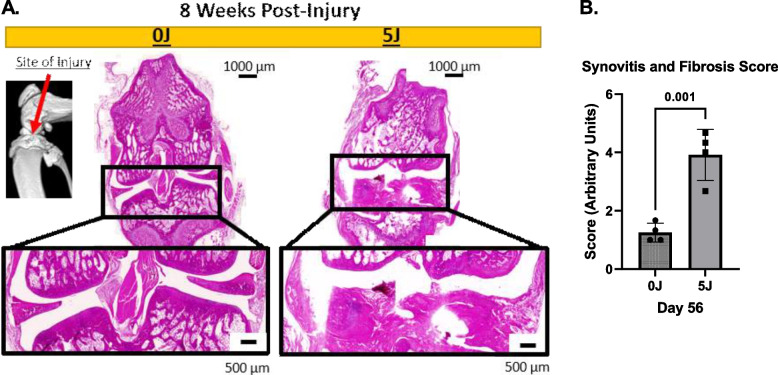
Histological Analysis of Impact Energies on IAF Healing. Representative H&E micrographs at 14 and 56 days post-injury from sham (0 J) and 5 J injury groups

**Fig. 7:**
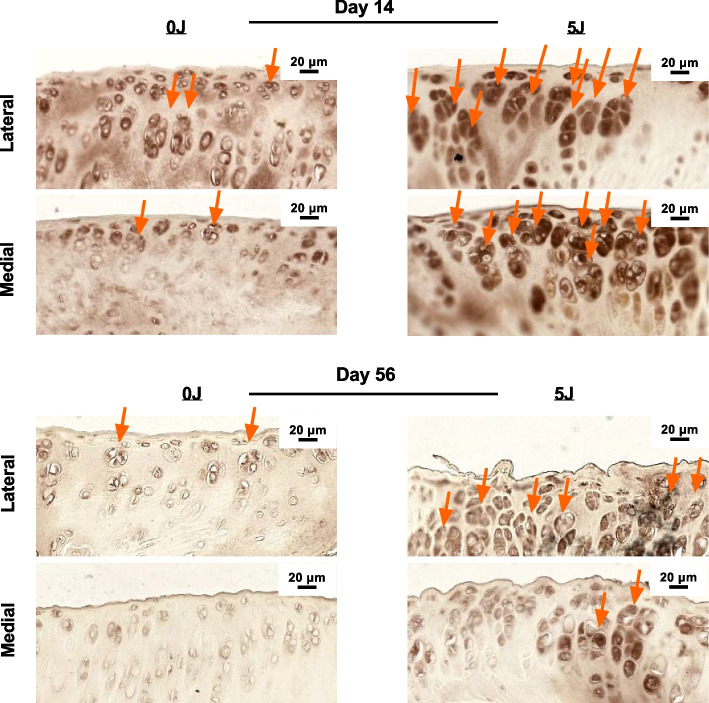
Immunohistochemical Analysis of Osteoclastogenic Bone Degradation Following IAF. Representative DCSTAMP micrographs at 14 and 56 days post-injury from sham (0 J) and 5 J injury groups

Qualitative assessment of the articular surface with H&E staining revealed that at Day 14 there was marked loss of bone architecture (Supplemental Fig. 3). At Day 56 (Fig. 6A) there is extensive cartilage breakdown with little to no articular cartilage remained in the 5 J group, compared to 0 J (sham) controls. Moreover, scoring the images revealed that in the 5 J group, there was reduced structural organization and marked fibrous tissue deposition at the articular surface concomitant to growth plate disruption compared to control (*P* < 0.012). In addition to H&E staining, OC were visualized at the articular surface of the 0 J and 5 J fractured tibias using surface marker DC-STAMP. DC-STAMP has been validated as a marker of OC differentiation as well as a biomarker for joint disease [16]. Qualitative assessment of ROI’s (Fig. 7) from the medial and lateral articular surfaces revealed differential OC expression between 0 and 5 J injuries. Briefly, at Day 14 (Fig. 7A) 0 J injuries resulted in no (medial) and few (lateral) OC versus 5 J were there were many OC could be seen (indicated by orange arrows). At Day 56 (Fig. 7B), no OC were observed in the 0 J group and less OC were observed in the 5 J group (compared to Day 14). However, the OC seen at Day 56 were larger and more diffuse along the bone.

## Discussion

The goal of the current study was to develop a blunt impact-based model of IAF in the rat and to characterize the degenerative PTOA pathobiology that is associated with such injuries as a means to establish a robust testbed for evaluating putative disease modifying therapies. We achieved this objective in that high energy impacts (5 J) to the lateral aspect of the knee consistently induce intra-articular, comminuted fractures of the tibial plateau that progressed to the pathological end-state of overt PTOA by 56 days post-injury. Additionally, this 5 J injury results in heightened levels of CCL2 in the SF of the affected knee, a finding that is consistent with clinical reports of elevated CCL2 levels in patients with knee OA [17, 18] and acutely following tibial plateau fracture [19]. CCL2 is particularly relevant in the context of osteoarthritis development following IAF as the CCL2/CCR2 signaling axis is a potent mediator both pain [20] and macrophage chemotaxis into the injured tissue. Furthermore, CCL2 is required to activate the repair response to chondral defects in mice [21]. This suggests that the persistent upregulation of CCL2 in the SF might be indicative of an ongoing process to sustain inflammation in an attempt to repair/regenerate the damaged articular surface in our IAF model.

In addition to inflammatory markers, COMP and NTX, markers of osteochondral degradation, have been directly implicated in both human PTOA pathology and various animal models of PTOA [22]. In the current model, we report that IAF increases circulating levels of COMP and NTX-1. Supporting these biochemical findings, micro-CT data showed that nearly all measures of bone morphometry in the tibial plateau and distal femur had a bone reduction when comparing the 5 J group to sham controls. Moreover, the morphological changes to trabecular structure following 5 J injury are similar to those reported in other models of PTOA, [23, 24] such that the 5 J injury described herein resulted in reduced trabecular number and increased trabecular thickness and separation compared to sham. It is unknown whether this response is fully attributable to the injury or if osteopenia plays a role as trabecular bone volume and mineral content have previously been shown to decrease with disuse of the limb in rats [25]. While further study of the relative contribution of these potentially superimposed effects on our IAF healing outcomes is warranted, this remains an important outcome in the model as these measures will serve to develop targeted therapeutics to improve overall joint health in future studies.

Finally, we were able to show, using routine histology, that the joint anatomy of the 5 J impact group is largely destroyed, and that the articular surfaces of the tibial plateau have been replaced with acellular fibrotic tissue. Moreover, the synovium appears to be hypercellular in the 5 J group relative to sham controls suggesting a chronic inflammatory state is associated with the observed tissue degradation, a striking observation that indicates a full joint pathology has taken hold. Additionally, and in support of the BMM findings (i.e. changes in bone volume), we showed an increase in the number of OC in the 5 J injured group at both Day 14 and 56 endpoints. These findings are in line with our observation of reduced overall bone volume associated with IAF injuries.

Together, these data can be used as a baseline model for screening putative disease-modifying therapeutics for mitigating the progression of PTOA. While other rodent models of PTOA described in the literature are likewise useful for such screening efforts, the model described herein may be particularly useful for evaluating interventions that might exhibit a ceiling effect in models on the lower end of the injury severity spectrum (e.g., destabilization of medial meniscus, ACL transection) as compared to models which truly replicate HELET. Similarly, if a candidate intervention shows efficacy in this model, one might postulate that it would also translate more favorably to higher-order species.

It is also important to note that the non-surgical aspects of the IAF induction described herein lends itself to further exploration of the early events that might occur following the injury. The work by Swart et al. [5] showed the impact of blunt articular impacts on chondrocyte viability increased with time over the first 72 h post-injury. In analogous surgical models, these types of effects might be obscured or unduly confounded by the inflammation and/or tissue damage associated with the surgical procedures required to induce the model. At the same time, however, while the non-invasiveness of our approach is beneficial for better understanding the basic pathobiology of IAF and the pathological processes that follow their occurrence, the lack of surgical reduction of these injuries is certainly a limitation of the current study. However, in addition to the technical difficulties associated with a small animal model including reduction and fixation (as typically follows HELET with IAF in humans), acceptable parameters for articular fracture reduction and hindlimb alignment have not been established in the rat knee. Additionally, while initial high-energy mechanisms of injury and initial fracture severity represent risk factors for PTOA in humans, fracture malreduction with persistent joint incongruity and, in particular for the knee joint, limb malalignment have greater influence on PTOA formation in both humans and large animals [26–29]. In our model, incongruity and resulting malalignment is simply allowed to persist and thus replicate these risk factors. This may have contributed to model reproducibility with regard to PTOA development, versus introducing an unwanted variable regarding the adequacy and stability of fracture reduction. Regardless, the most prudent immediate use of this model system would be for early screening and mechanistic studies focused on the mitigation of PTOA pathobiology that can be subsequently confirmed in an analogous large animal system wherein such surgical reduction can be reliably performed.

## Conclusions

We have developed herein a robust and reproducible small animal model that could be useful for evaluating therapeutics aimed at mitigating the degenerative processes that follow high severity IAF and often lead to end-stage osteoarthritis. This model recapitulates the critical hallmarks of clinical OA including radiologic changes, local inflammatory markers, systemic osteochondral degradation markers, and histological findings that could serve as key measurements for efficacy assessments. Given the inherent lack of surgical reduction in this non-invasive injury model, this model will be most valuable as an early screening tool in a coordinated translational research pipeline such that promising results in this rat model and be efficiently confirmed in an analogous large animal model wherein surgical reduction can be reliably and reproducibly performed.

## Supplementary Information

**Additional file 1.**

**Additional file 2. **

**Additional file 3. **

## Data Availability

All data generated or analyzed during this study are included in this published article and its supplementary information files.
