# Supplementary figures and images for: Development and characterization of an intra-articular fracture mediated model of post-traumatic osteoarthritis
Source: J Exp Orthop. 2023 Jul 4;10:68. doi: 10.1186/s40634-023-00625-9 (PMC10317931; doi:10.1186/s40634-023-00625-9)

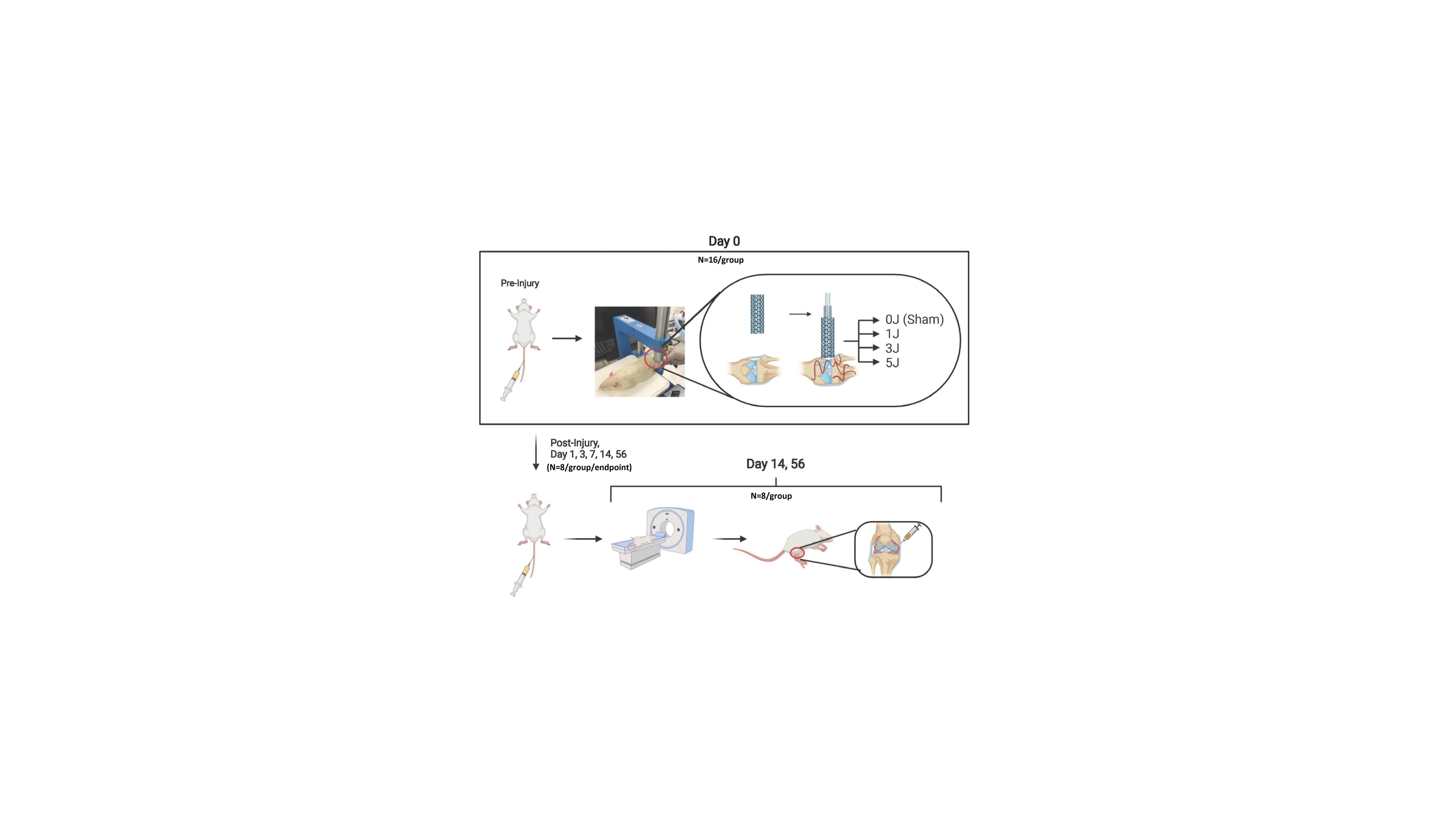

Supplement: Supplementary file 1 — Additional file 1. [file 40634_2023_625_MOESM1_ESM.tiff]

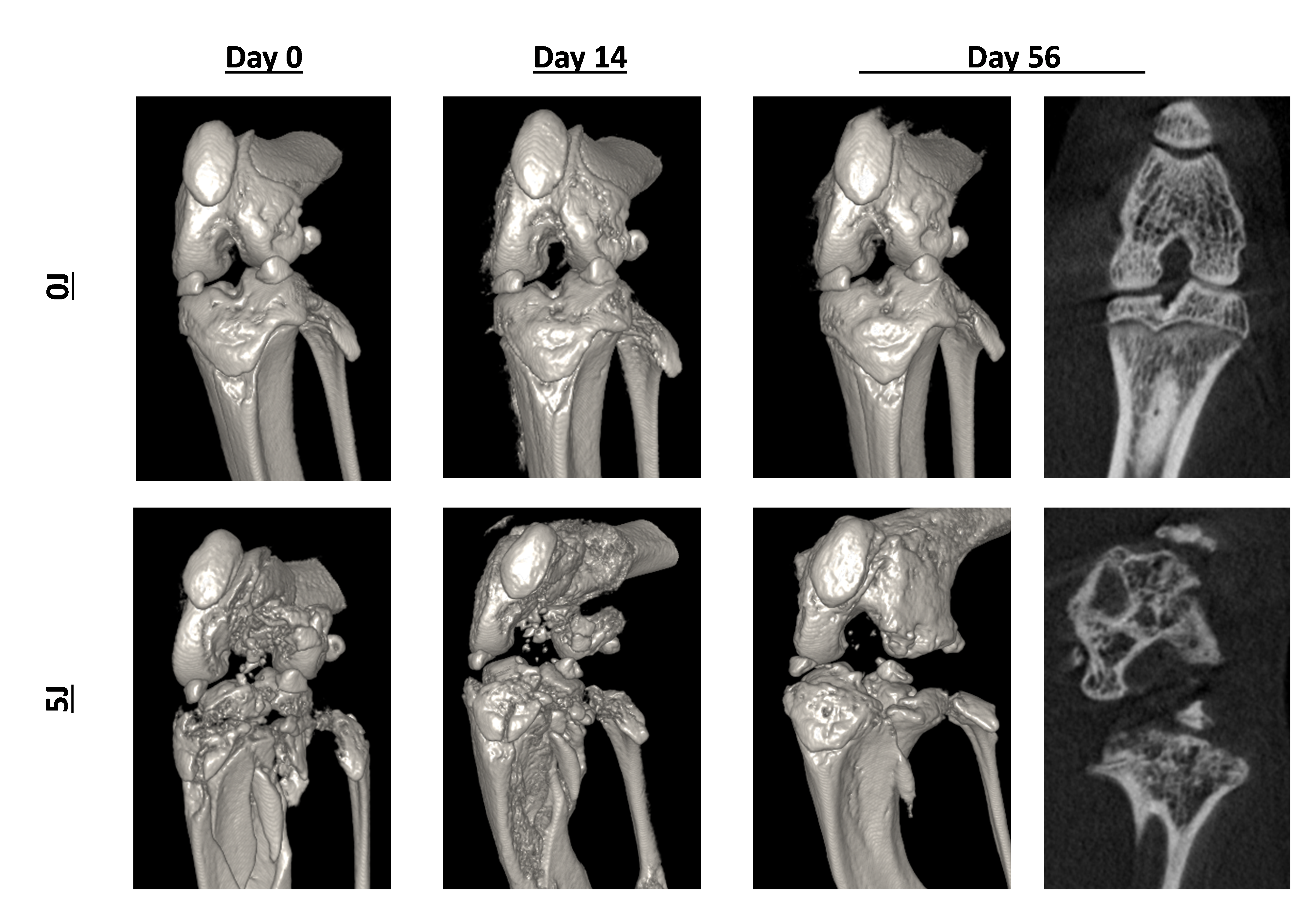

Supplement: Supplementary file 2 — Additional file 2. [file 40634_2023_625_MOESM2_ESM.tif]

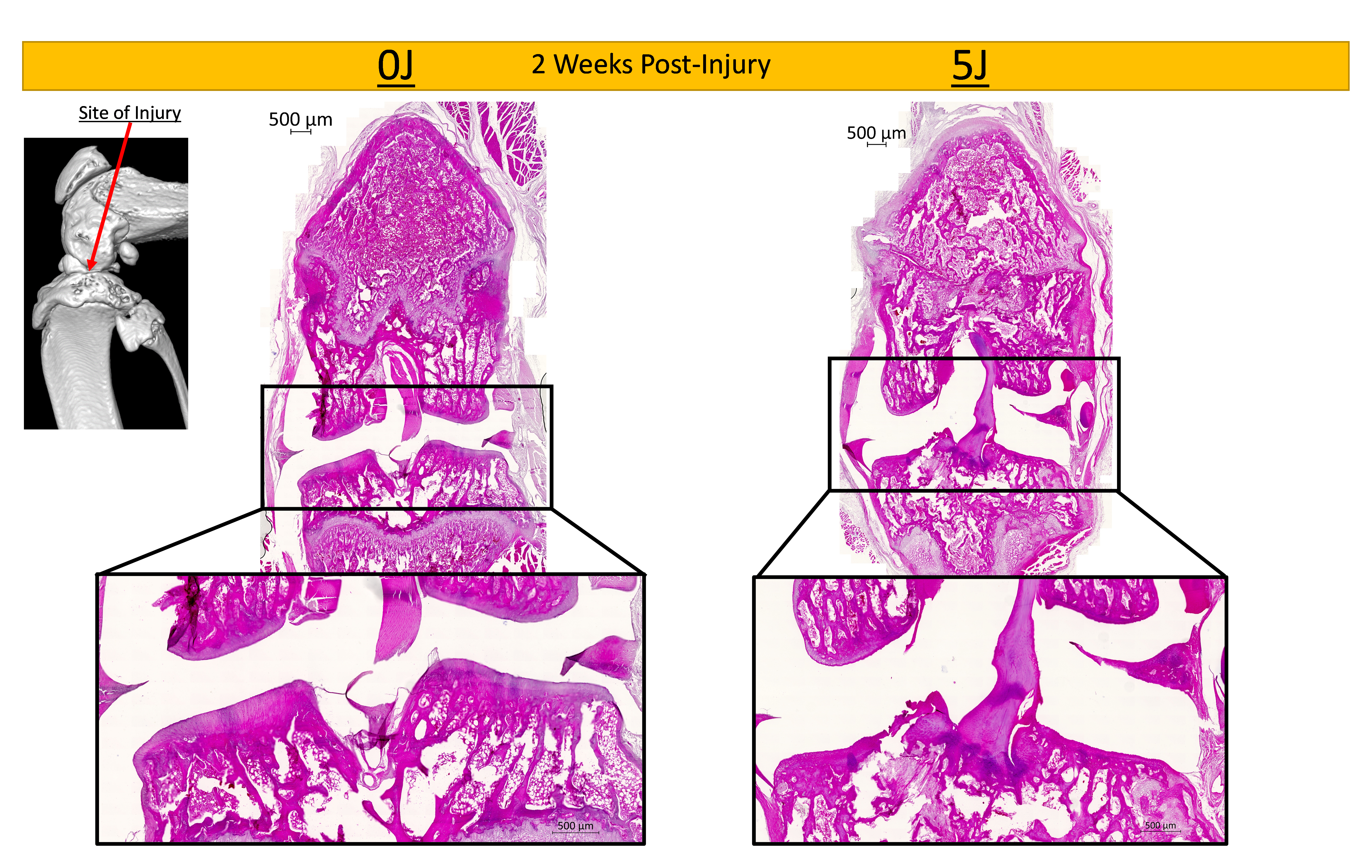

Supplement: Supplementary file 3 — Additional file 3. [file 40634_2023_625_MOESM3_ESM.tif]
